# Algorithmic Compensation of the Polarization Cross-Sensitivity of TFBG Sensors for SRI Measurement

**DOI:** 10.3390/s26216836

**Published:** 2026-10-29

**Authors:** Sławomir Cięszczyk, Krzysztof Skorupski, Patryk Panas, Jacek Klimek, Damian Harasim

**Affiliations:** Department of Electronics and Information Technology, Lublin University of Technology, Nadbystrzycka 38A, 20-618 Lublin, Poland; k.skorupski@pollub.pl (K.S.); p.panas@pollub.pl (P.P.); j.klimek@pollub.pl (J.K.); d.harasim@pollub.pl (D.H.)

**Keywords:** TFBG, refractive index, demodulation algorithm, optical fiber sensor

## Abstract

Tilted fiber Bragg gratings are sensitive to a range of physical quantities, which unfortunately gives rise to undesirable cross-sensitivities. In refractive index measurements, the polarization of the radiation introduced into the optical fiber is an additional factor affecting the measurement. Analysis of optical spectra containing several dozen resonances mainly involves extracting features from the spectrum and constructing a regression model relating these features to the refractive index. In this article, we propose the use of a shallow artificial neural network as the regression model. The shifts in several dozen cladding modes were used as input features for the model. Additional features included the cutoff wavelength and the spectrum length, both of which were determined from the spectra. We demonstrated that the sensitivity of refractive index determination to polarization is reduced compared with classical methods such as the cutoff wavelength method.

## 1. Introduction

Fiber-optic sensors are widely used in many different fields of science and technology. Tilted fiber Bragg gratings (TFBGs) can be used to measure quantities such as the angle of rotation, bending radius, temperature and stress. However, they are most promising for measuring the surrounding refractive index (SRI) of liquids. Unfortunately, during measurement, either the polarization of the incident radiation must be kept constant or a suitable compensation method must be employed. One hardware-based compensation method uses the Perpendicular TFBG (P-TFBG) system consists of two TFBGs inscribed with a 90° angular rotation relative to each other around the central axis of the fiber [1]. Different configurations of multiple TFBGs incorporated into a single optical fiber can be designed to optimize specific measurement characteristics. For example, the measurement range can be extended by employing TFBGs with different tilt angles [2], while the SRI measurement resolution can be enhanced through the use of TFBGs with slightly shifted spectra [3] or inserting fiber in capillary [4]. Algorithmic compensation may utilize polarization information based on a portion of the spectrum that is less sensitive to the refractive index of the liquid (SRI). However, for TFBGs, a polarization change affects the entire range of cladding modes [5]. Determining the SRI value requires analyzing the cladding mode spectrum using digital signal processing algorithms.

Wavelength tracking methods utilize both local and global spectral features. Local methods make use of shifts in the resonance wavelengths associated with individual cladding modes within a narrowband spectral shift [6]. Global methods involve the analysis of the properties of multiple cladding modes over a broad spectral range [7,8]. One possible approach is based on tracking the cutoff wavelength [9]. The global wavelength tracking method is characterized by a linear relationship between the cutoff wavelength shift and SRI, whereas for local methods, this relationship is highly nonlinear.

However, when the refractive index and polarization state vary simultaneously, the wavelength shifts of individual cladding-mode resonances can exhibit complex, nonlinear dependences on both quantities. Such relationships can be modeled using nonlinear machine learning methods. Artificial neural networks have been used to demodulate various types of fiber-optic sensors [10,11]. Machine learning has also been applied to TFBG-based sensors [12], with Gaussian process regression, Random Forest, k-nearest neighbors, AdaBoost, and Gradient Boosting evaluated for simultaneous temperature and humidity estimation. Gradient Boosting achieved the best performance among the methods investigated. In another study [13], the input features included the wavelength shift of a selected cladding-mode resonance, the change in transmission at that resonance, the number of spectral minima, the wavelength of the envelope minimum, the change in transmission at the envelope minimum, and the area associated with the cladding-mode spectrum. These features were used in a k-nearest neighbors (kNN) regression model. A densely connected convolutional neural network has also been employed for demodulation [14]. In this approach, the transmission spectrum is fed directly into the network without preprocessing. Successive layers extract features from the input data and concatenate them with features obtained from preceding layers. Preprocessing-free demodulation has also been demonstrated using convolutional neural networks (CNNs) with residual blocks [15]. However, this architecture is relatively complex and comprises multiple layers. The use of machine learning aims to improve measurement performance, including accuracy and resolution, while enabling algorithmic compensation for cross-sensitivity effects. The main challenge associated with using deep neural networks is the large training dataset required during the training phase. Only with such a dataset can the model achieve an adequate level of generalization. A second challenge is that the TFBG spectrum is analyzed independently of its physical properties. Replacing the entire spectrum with a set of extracted spectral parameters reduces the dimensionality of the model input vector, allowing for a substantially simpler model and, consequently, a faster training process. Moreover, the parameters derived from the spectrum, such as the resonance wavelength shifts of individual modes, represent physical quantities that are directly related to the SRI value. Similarly, the cutoff wavelength is a physically meaningful parameter. The spectral length also provides information related to the total intensity of the set of cladding modes.

In this article, we propose a new algorithmic method for reducing the influence of polarization on SRI estimation by analyzing the wavelength shifts of multiple cladding-mode resonances across the full cladding-mode spectral range. Joint analysis of multiple modes can improve parameter estimation and support temperature compensation [16]. Simultaneous measurement of strain and temperature may require an additional optical element alongside the TFBG, such as a microfiber interferometer (MFI) [17]. However, methods have also been reported for simultaneously determining temperature and strain solely from the TFBG spectrum [18]. Analyzing multiple cladding-mode resonances rather than a single resonance can improve detection performance [19,20]. Mapping the wavelength shifts of several dozen resonances to SRI reduces estimation errors caused by changes in polarization. The method was evaluated using both a single TFBG and a PTFBG configuration with reduced polarization sensitivity.

## 2. Materials and Methods

### Optical Spectrum Measurements and Analysis of Cladding Mode Shifts

**Figure 1:**
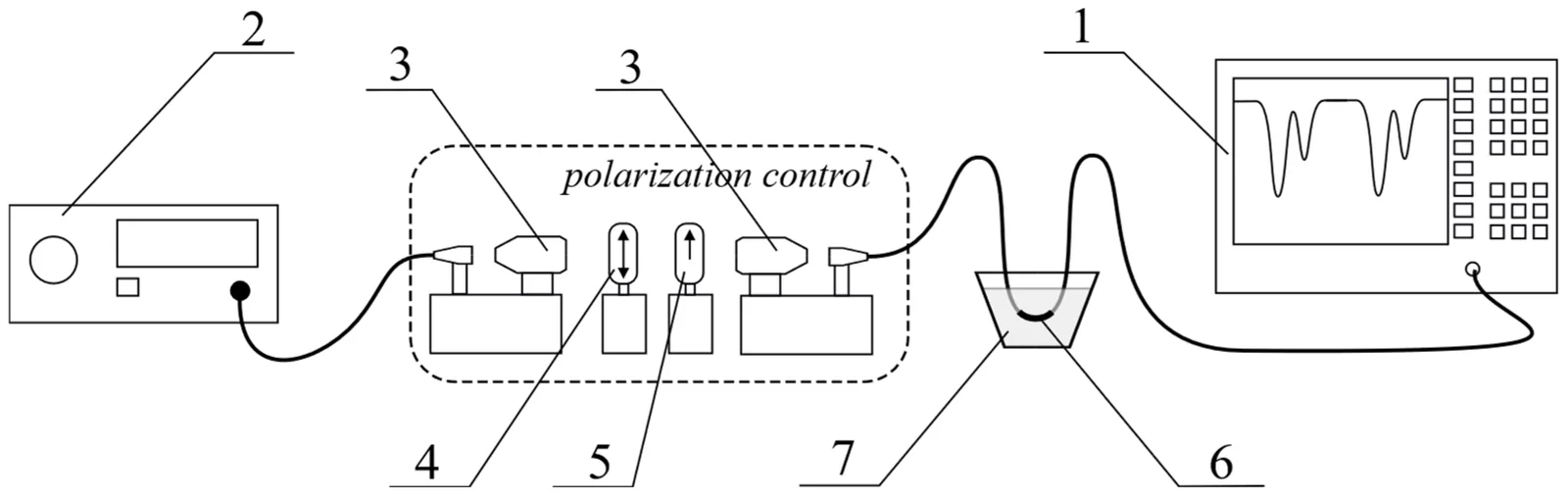
Measurement setup enabling simultaneous adjustment of the input light polarization and the refractive index of the test solution.

The measurement system, which consists of a 1–optical spectrum analyzer AQ6380 (Yokogawa Electric Corporation, Kanagawa, Japan), 2–broadband SLED source, 3–objectives, 4–polarizer, 5–half-wave plate in an electronically driven rotation stage, 6–container for the analyzed solution, and 7–TFBG/P-TFBG sensor, is shown in Figure 1.

Solutions with target surrounding refractive index (SRI) values were prepared by mixing water and cane sugar in specified proportions. The refractive index of each solution was independently measured using a laboratory refractometer with an accuracy of 10^−5^ refractive index units (RIU), and these measurements served as reference values. The temperature was maintained at 23 °C throughout the measurements. The polarization angle was varied using a half-wave plate. Since rotating the half-wave plate by 1° rotates the polarization direction by 2°, the plate was rotated in 1° steps to obtain polarization-angle increments of 2°. The half-wave plate was mounted on a Thorlabs (Thorlabs Inc., Newton, NJ, USA) PRM1Z8 motorized rotation stage with a specified bidirectional repeatability of ±0.1°. A polarization angle of 0° was defined as the P-polarized state, with the electric field oriented parallel to the grating tilt plane.

**Figure 2:**
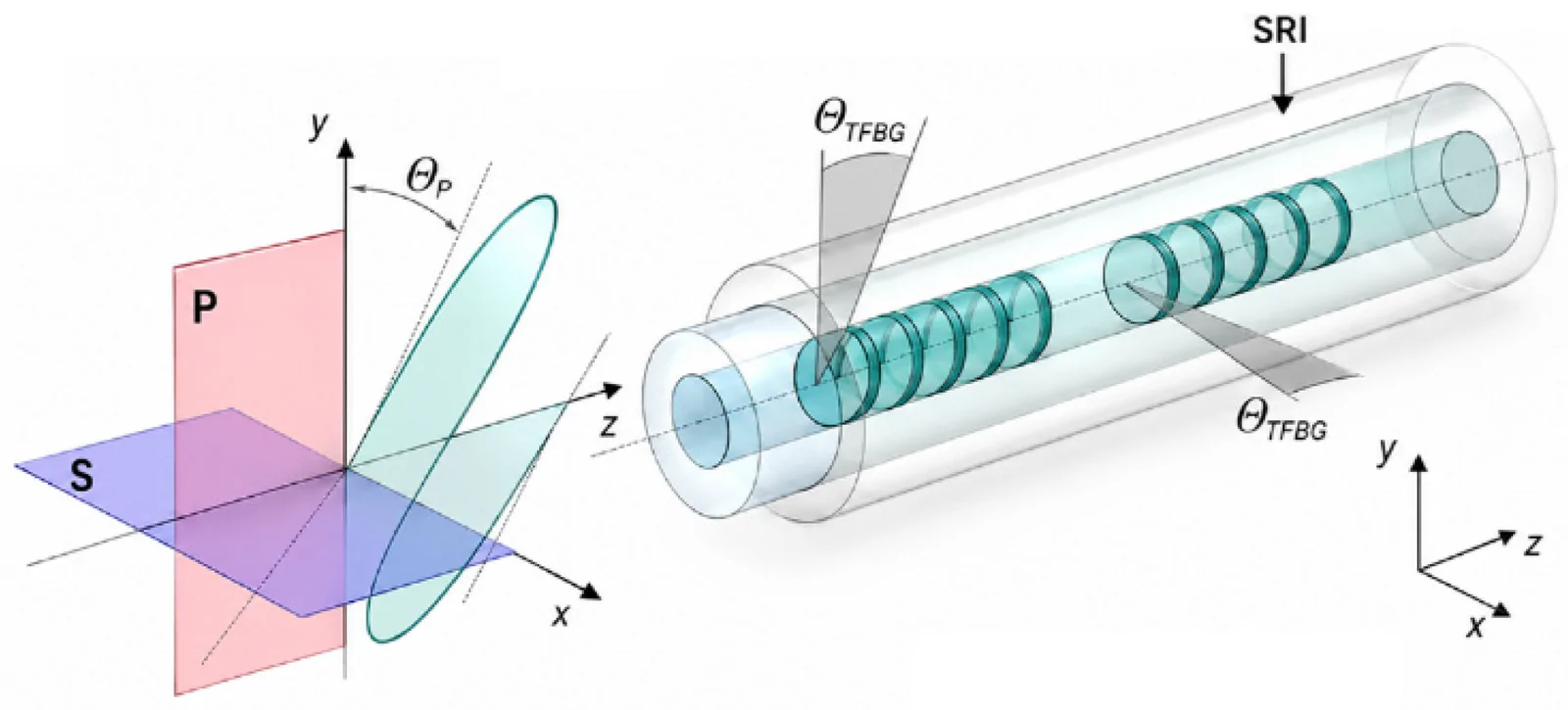
Schematic view of the internal structure of a PTFBG.

An integral structure of the PTFBG sensor, which consists of two TFBGs with precisely matched spectral parameters of cladding modes, is shown in Figure 2.

**Figure 3:**
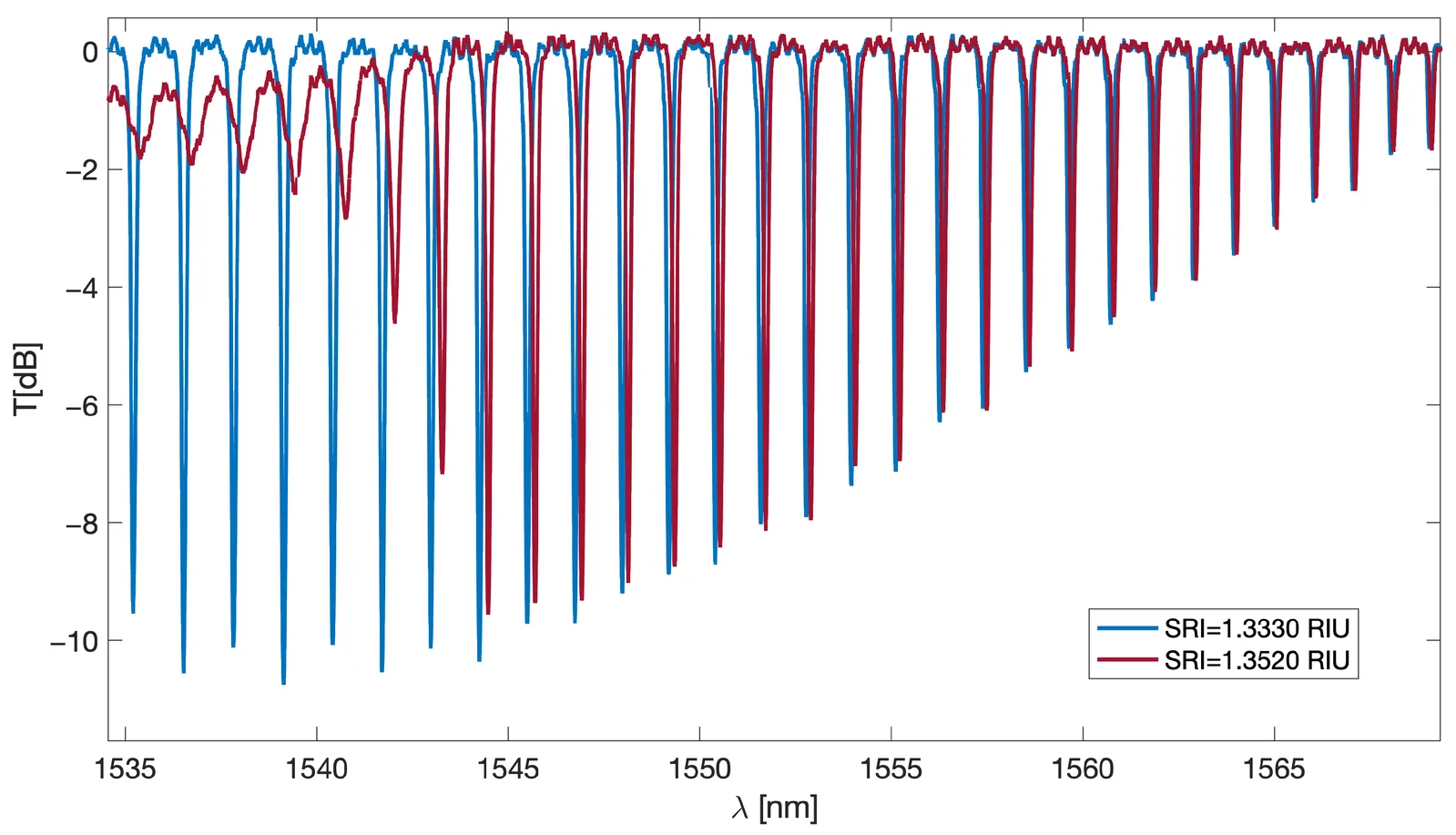
Spectrum of the analyzed TFBG grating for two extreme SRI values.

Measurements were taken at 13 SRI values and 46 polarization angles, resulting in a total of 598 optical spectra. Each acquired spectrum contained 22,501 data points covering the wavelength range from 1490 nm to 1580 nm, with a spectral resolution of 0.004 nm. The SRI values were varied using mixtures of cane sugar and hydrogen peroxide, while the actual SRI values were measured using a reference method with an accuracy of 10^−5^ RIU. The measured SRI values ranged from 1.3330 to 1.3403 RIU. The measurements were performed under stable laboratory temperature conditions. As shown in Figure 3, cladding mode shifts were observed in the transmission spectra because of changes in the SRI.

## 3. Results

**Figure 4:**
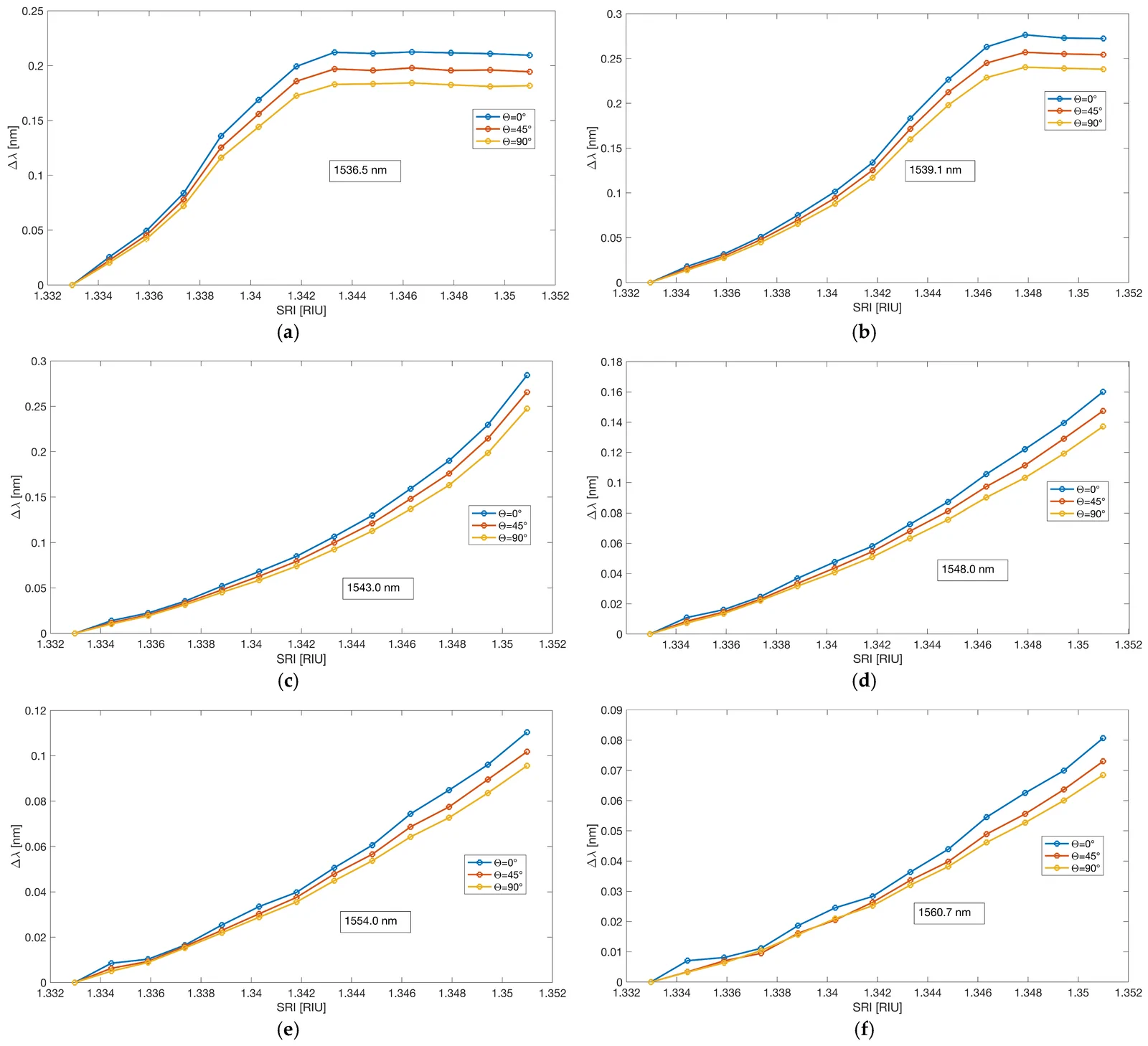
Shift in the resonance wavelength of: (**a**) 1536.5 nm; (**b**) 1539.1 nm; (**c**) 1543.0 nm mode; (**d**) 1548.0 nm mode; (**e**) 1554.0 nm and (**f**) 1560.7 nm, as a function of SRI for three polarisation values.

Figure 4 shows the changes in the estimated wavelengths of selected cladding-mode resonances as a function of SRI for three polarization angles. The angles of 0° and 90° correspond to orthogonal polarization states. The responses shown in Figure 4a,b differ in shape from those presented in Figure 4c–f. The resonances near 1538.5 nm and 1539.1 nm initially shift towards longer wavelengths as SRI increases. Above a certain SRI value, however, their estimated wavelengths become approximately constant despite further increases in SRI. The resonance near 1538.5 nm reaches this saturation region at a lower SRI than the resonance near 1539.1 nm. This behavior is consistent with the transition from guided to leaky cladding modes as the cutoff wavelength shifts towards longer wavelengths with increasing SRI. A cladding mode becomes leaky when its effective refractive index falls below that of the surrounding medium, placing its resonance on the shorter-wavelength side of the cutoff. In this regime, further increases in SRI primarily reduce the resonance depth, while its wavelength remains approximately constant. The separation between the curves corresponding to different polarization angles in Figure 4a,b increases with SRI and is particularly pronounced in the saturation region. These polarization-dependent differences in the estimated resonance wavelengths introduce errors into SRI determination based on individual cladding-mode resonances. Moreover, the reduced wavelength sensitivity in the saturation region makes SRI estimation especially susceptible to such deviations. To determine the resonance wavelengths, the transmission spectrum was first expressed on a logarithmic scale, after which the resulting values were squared. The wavelength of each selected resonance was then estimated using the centroid method within its corresponding spectral window.

A mode with a wavelength of 1543 nm (Figure 4c) does not become a leaking mode within the range of SRI values under consideration. As the SRI value increases, the divergence between the curves for different polarisations increases. However, this divergence is smaller than in the case of modes that have exceeded the cut-off wavelength and are leaking out of the optical fibre. For each subsequent mode, which is situated at longer wavelengths, the mode shift decreases as the SRI increases. This implies that subsequent modes are less sensitive to changes in the refractive index. At the same time, the sensitivity of subsequent modes (Figure 4d–f) to polarisation decreases slightly. It should be noted that there is significant non-linearity in the relationship between the mode wavelength shift and the SRI, as well as between the wavelength shift and polarisation.

**Figure 5:**
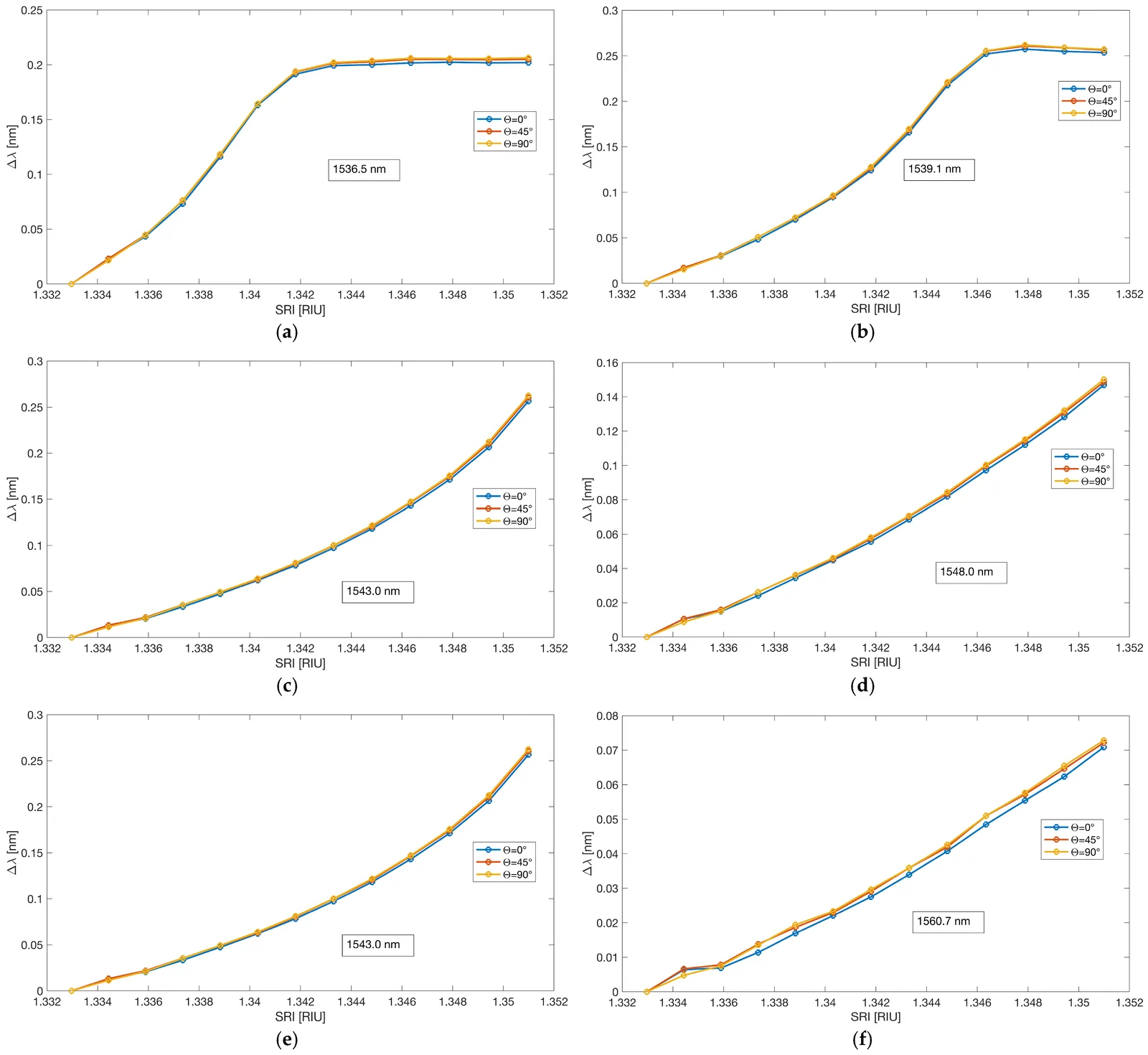
Shift in the mode at wavelength of: (**a**) 1536.5; (**b**) 1539.1; (**c**) 1543.0 nm; (**d**) 1548.0 nm; (**e**) 1554.0 nm; (**f**) 1560.7 nm as a function of SRI for three polarisation values, for PTFBG grating array.

Figure 4a–f presented below show graphs analogous to Figure 5a–f, but they present measurements for the PTFBG grating array. In each of these figures, compared with the previous figures for TFBG, there are noticeably smaller differences between the individual curves. However, the curves do not coincide perfectly; therefore, cross-polarisation sensitivity will not be completely eliminated.

### 3.1. Determining the Cut-Off Wavelength Shifts

**Figure 6:**
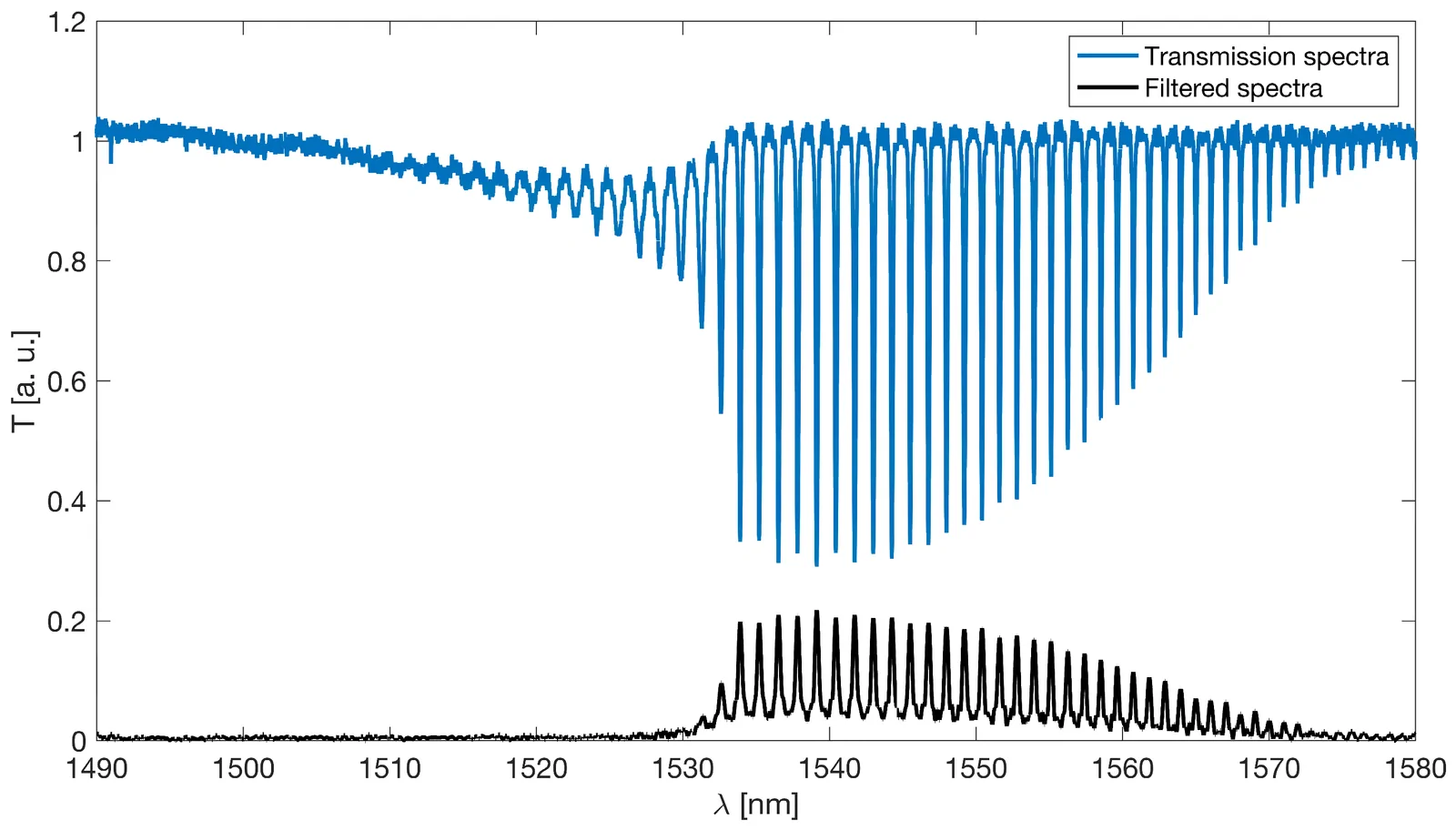
The transmission spectrum of a TFBG grating and the same spectrum after Fourier filtering.

Determining the cut-off wavelength shifts typically requires identifying the point at which the cladding modes’ intensity changes the most. Analysing the optical spectrum consists of several steps. First, Fourier filtering is used to determine the frequency at which the cladding modes occur. Next, the absolute value of the filtered spectrum is calculated. Figure 6 shows an example of a TFBG transmission spectrum and its filtered version. The curve shows the spectrum for a refractive index value of 1.3330 RIU.

**Figure 7:**
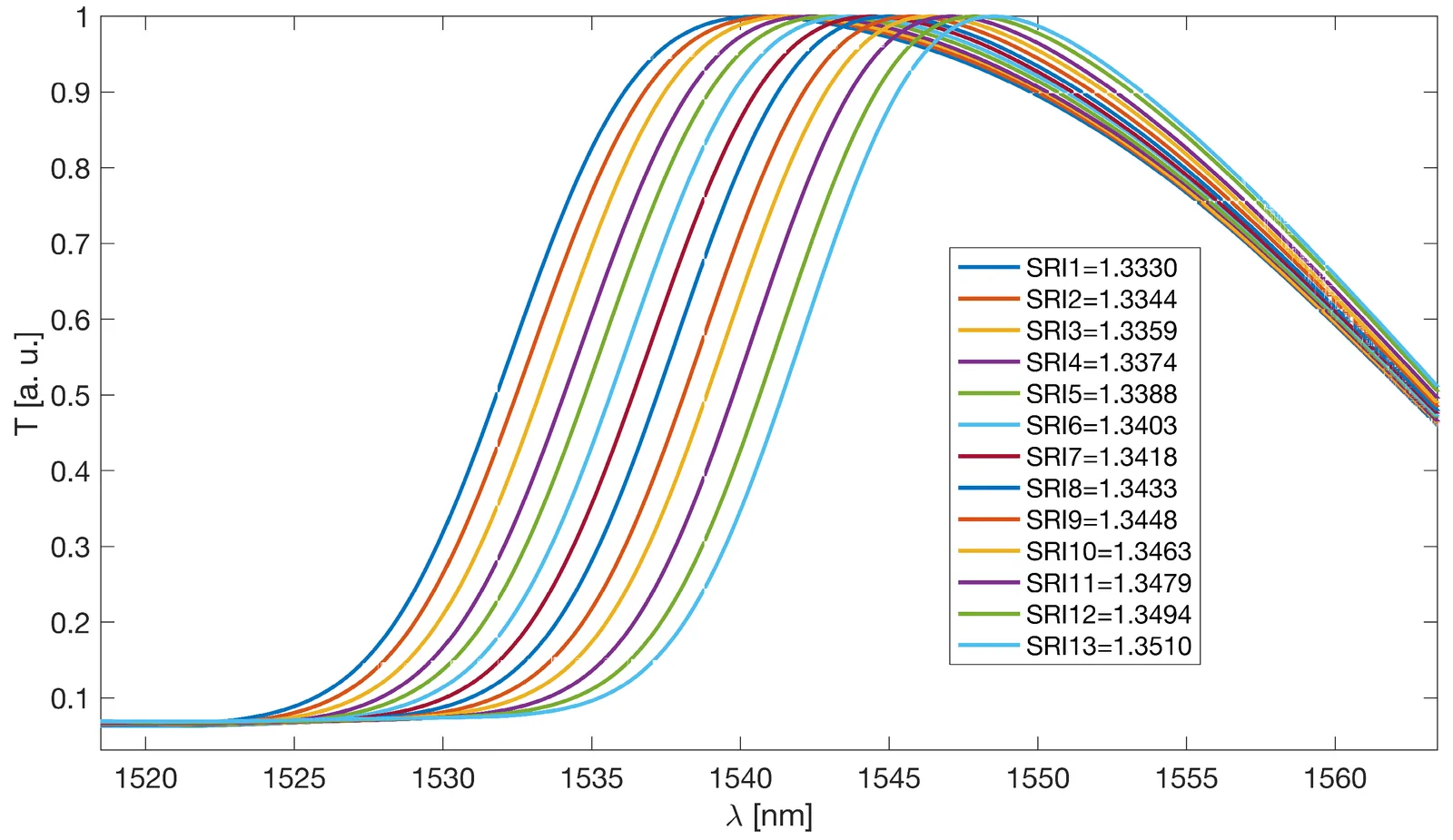
The smoothed transmission spectrum of the TFBG grating following Fourier-domain filtering.

**Figure 8:**
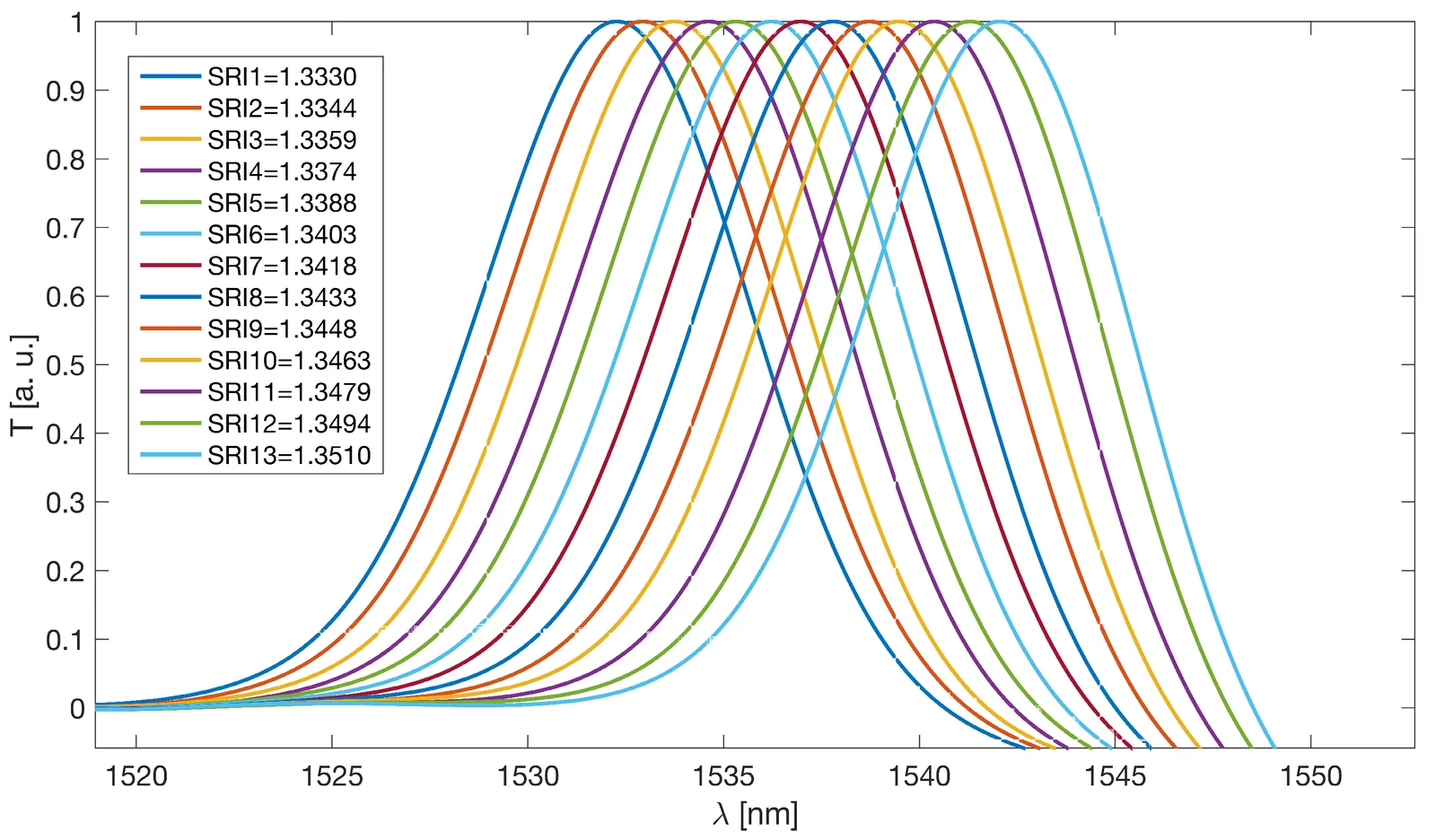
Normalized derivatives of the spectra.

The filtered spectrum must be smoothed thoroughly. In our case, we applied a Gaussian impulse response filter four times. The smoothed and normalised spectrum is shown in Figure 7. This contains the spectra of all 13 of the measured SRI values for the same polarisation. The derivatives of these spectra are shown in Figure 8. The derivatives were calculated using the first-order finite difference method. Furthermore, the resulting spectra were normalised to facilitate comparison of their shifts in response to changes in SRI. The normalization of the curves was performed by dividing all values of each curve by its maximum value. The shift in the maximum values of the curves in Figure 8 corresponds to a shift in the cut-off wavelength. This shift can be determined using the centroid method.

**Figure 9:**
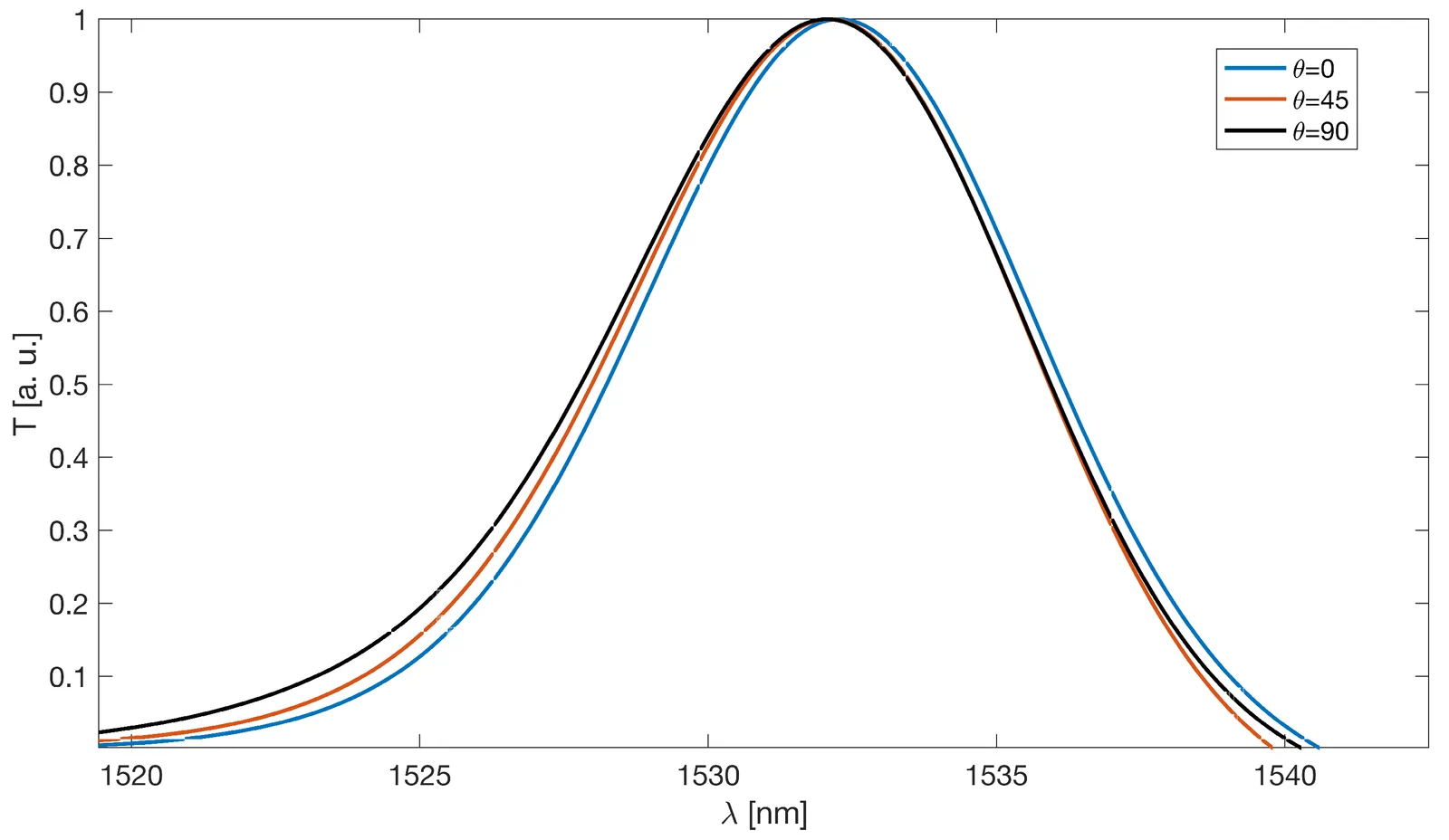
Derivatives of spectra from Figure 8 for a single SRI value and three polarisations.

If the curves in Figure 8 were identical for all polarisation values and a given SRI value, polarisation would have no effect on the determination of the SRI. Figure 9 shows how the curve in Figure 8 changes under the influence of polarisation for a single SRI value.

**Figure 10:**
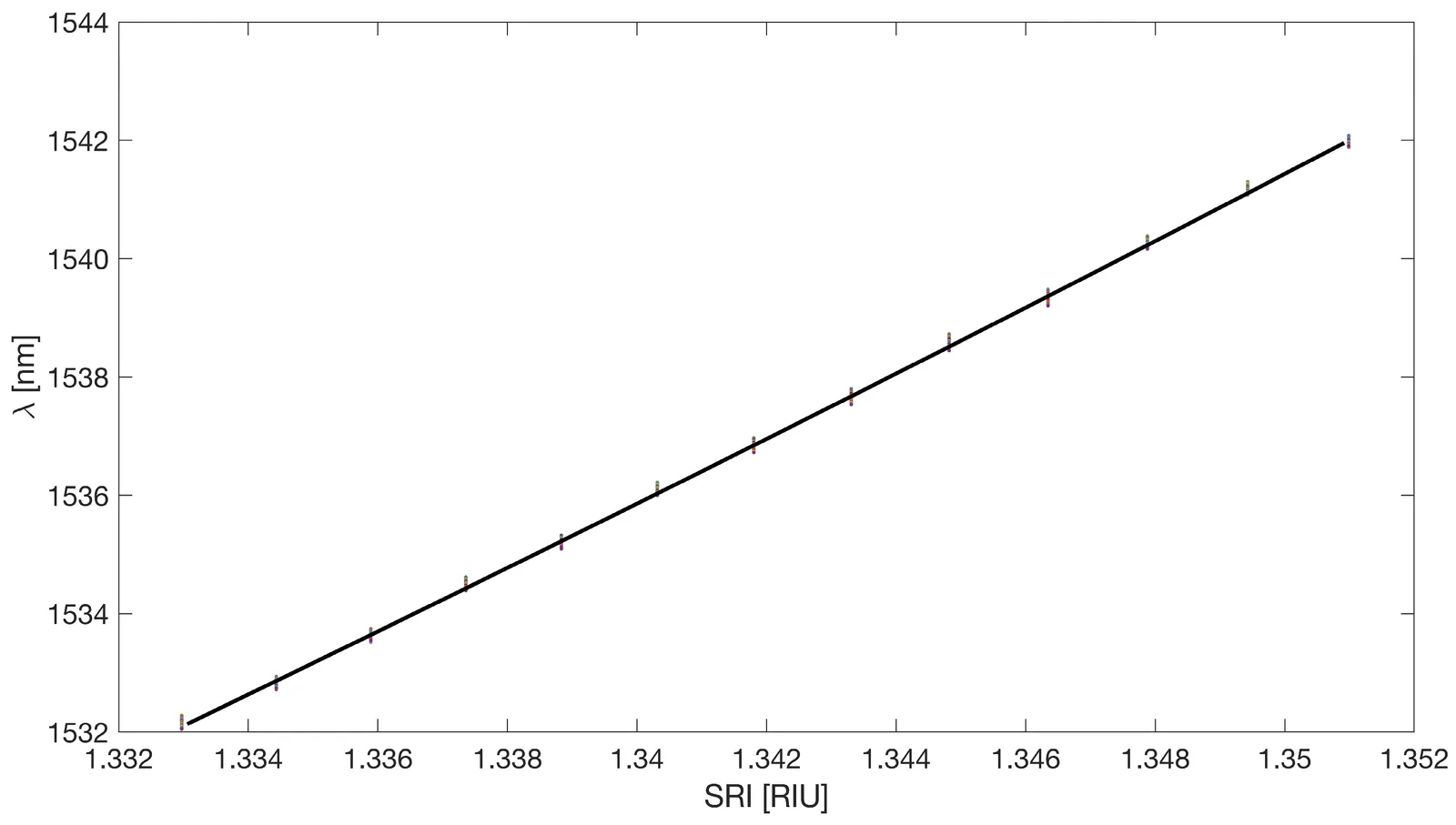
The relationship between the cut-off wavelength and the SRI.

**Figure 11:**
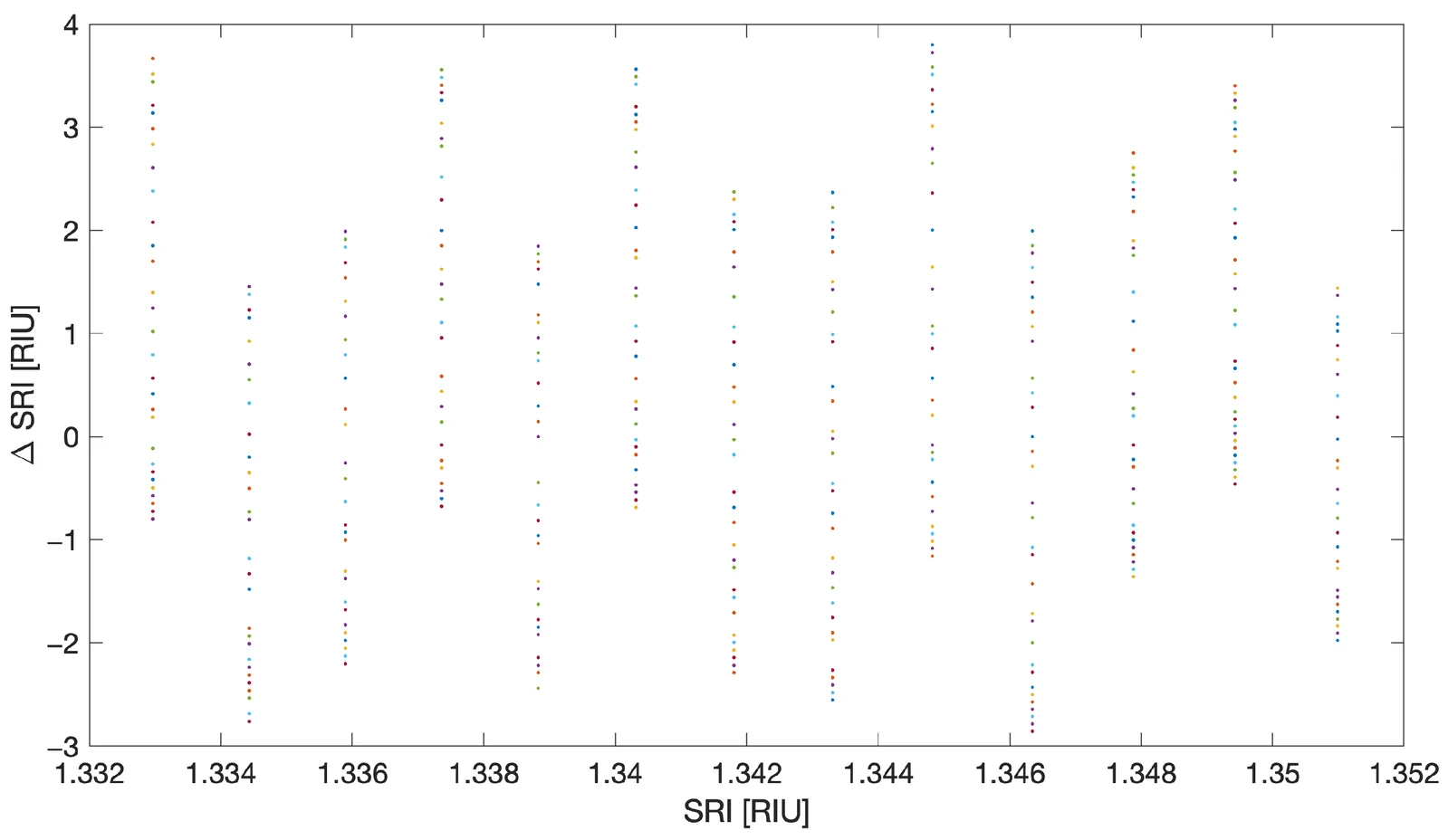
Errors in determining the SRI using a method based on the cut-off wavelength shifts.

Figure 10 shows the determination of the cut-off wavelength for individual SRI values and for all polarisations. The cut-off wavelength is the maximum of the curves in Figure 8, taking into account their variation with polarisation (Figure 9). The SRI values were determined on the basis of the calibration curve in Figure 10 and the individual cut-off wavelength values. Figure 11 shows the differences between the reference SRI values and those determined on the basis of the cut-off wavelength. The scatter of the individual error values indicates the influence of polarisation on the determination of SRI using the cut-off wavelength method.

### 3.2. Correction of Cross-Sensitivity Due to Polarisation Using a Neural Network

**Figure 12:**
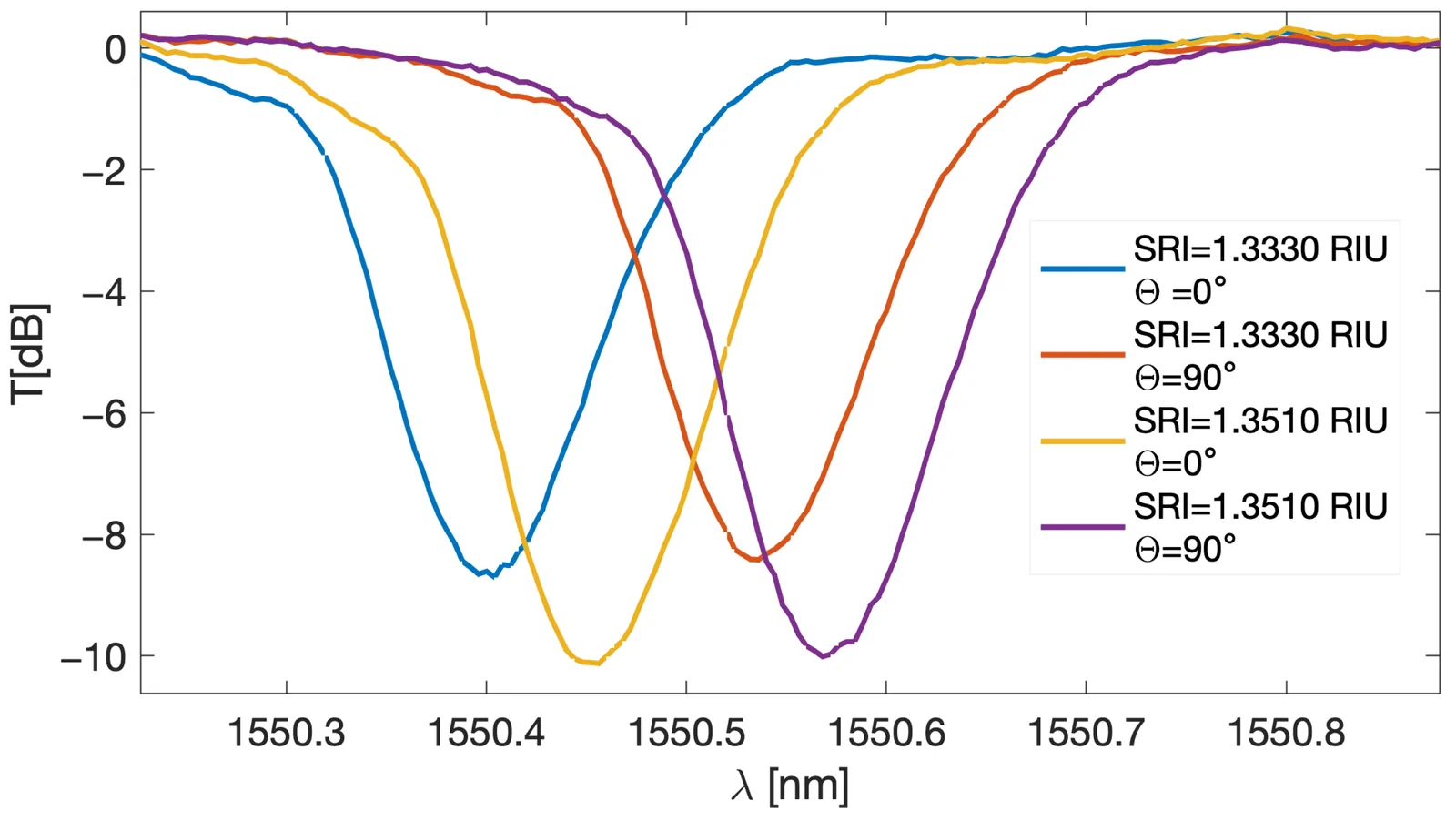
The spectrum of a single mode of the TFBG grating for two extreme SRI values and two extreme polarizations.

The mode shifts are nonlinear. Furthermore, once the cutoff wavelength is exceeded, the modes cease to shift and begin to decrease in amplitude. The SRI value can be determined from the shift of a single mode using a nonlinear relationship in the form of a polynomial regression. However, changes in polarization result in additional mode shifts (see Figure 12) and associated errors. Owing to individual modes’ varying sensitivity to SRI and polarization, it is preferable to use information from the shifts of multiple modes. An artificial neural network (NN) is the preferred choice for creating such a model. However, it does not need to be a highly complex deep network. In our research, we used a feedforward neural network consisting of three layers. The input layer contains 20 neurons, the same number as the modes used to determine the shift. There is a single hidden layer containing 15 neurons and a single output neuron, as only a single refractive index value is determined. Each neuron in a layer is connected to every neuron in the next layer. During the training phase, we used the Bayesian Regularization algorithm, which proved to be more effective than the popular Levenberg–Marquardt algorithm. The hidden layer used the hyperbolic tangent sigmoid activation function, while the output layer used a linear activation function. These functions were fixed; no activation-function search was performed. The maximum number of training epochs was set to 1000, and the minimum-gradient threshold was set to 10^−7^. Training terminated after 13 epochs because the minimum-gradient stopping criterion was reached. No predefined range of regularization coefficients was searched. Instead, the training algorithm automatically adapted the coefficients balancing the sum of squared training errors and the sum of squared weights and biases during optimization. The shifts of the 20 most sensitive modes were used as the input vector. The hidden layer contains 15 neurons, as increasing their number did not reduce the SRI estimation errors. Conversely, reducing the number of neurons to 10 resulted in an increase in the SRI estimation errors. Next, for the 15 neurons in the hidden layer, the effect of the input vector length was analysed. This involves selecting the modes whose shifts will be used to estimate the SRI. Similar RMSE values were obtained for the number of modes ranging from 15 to 25. It is important, however, that the input data contain modes that, for higher SRI values, exhibit leakage, such as the mode with an initial wavelength of 1536.5 nm.

In the first stage, randomly selected measurement points were used for training, testing and validation. These subsets accounted for 80 percent, 10 percent and 10 percent of the entire spectrum dataset, respectively. To characterize the properties of the proposed demodulation method, we present two parameters: the root mean square error (RMSE) and the Standard Deviation (SD) of the SRI estimates based on TFBG spectrum measurements. Lower values of both parameters indicate a better method with lower sensitivity to changes in polarization. For each SRI, we estimate the root mean square error (RMSE) value from 46 measurements for different polarizations:

(1) $$RMSE=\sqrt{\frac{1}{46}\sum_{i=1}^{46}{\left({SRI}_{i}-{SRI}_{true}\right)}^{2}}$$

**Figure 13:**
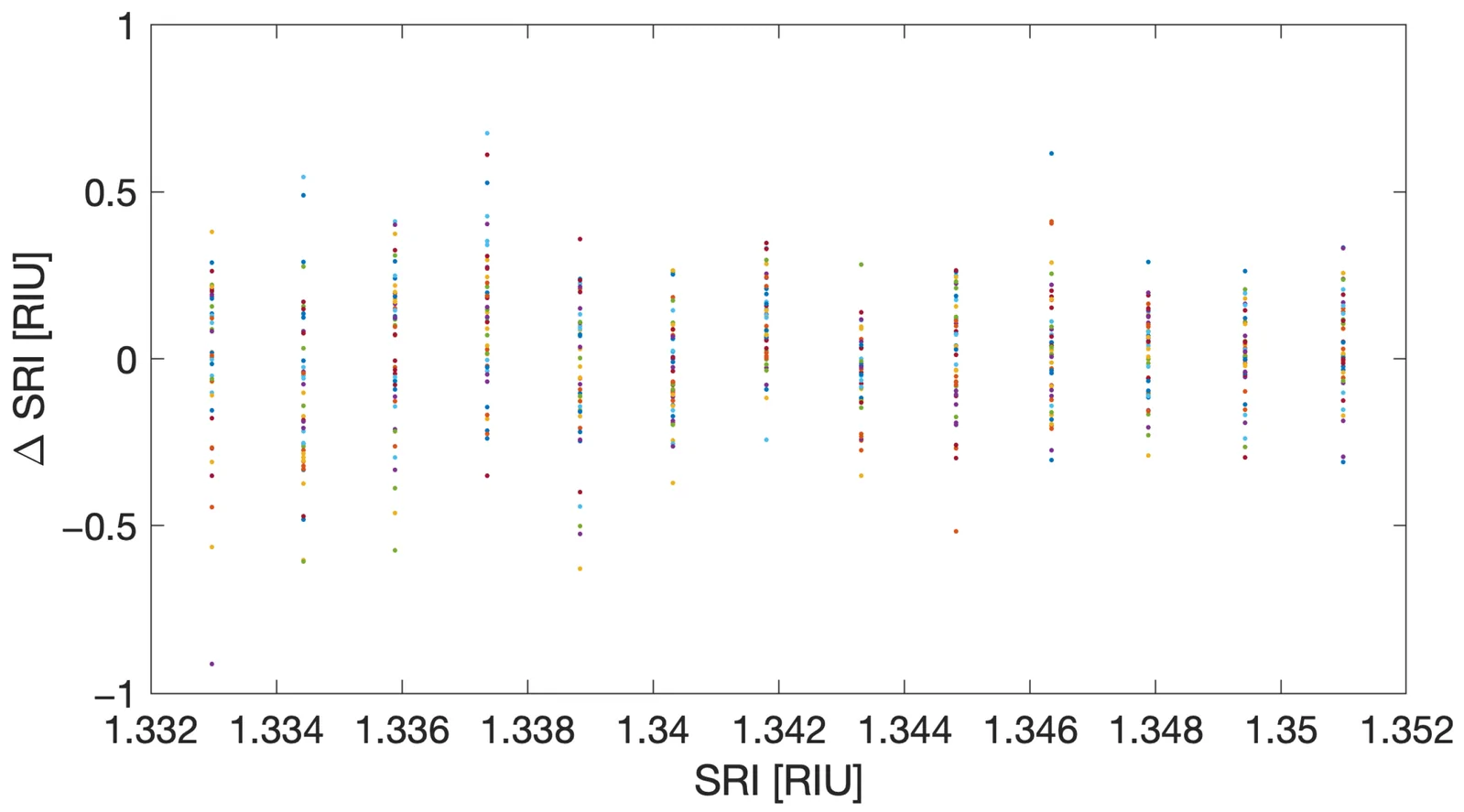
Errors in determining SRI values for all measurement points in the case of the TFBG grating.

**Figure 14:**
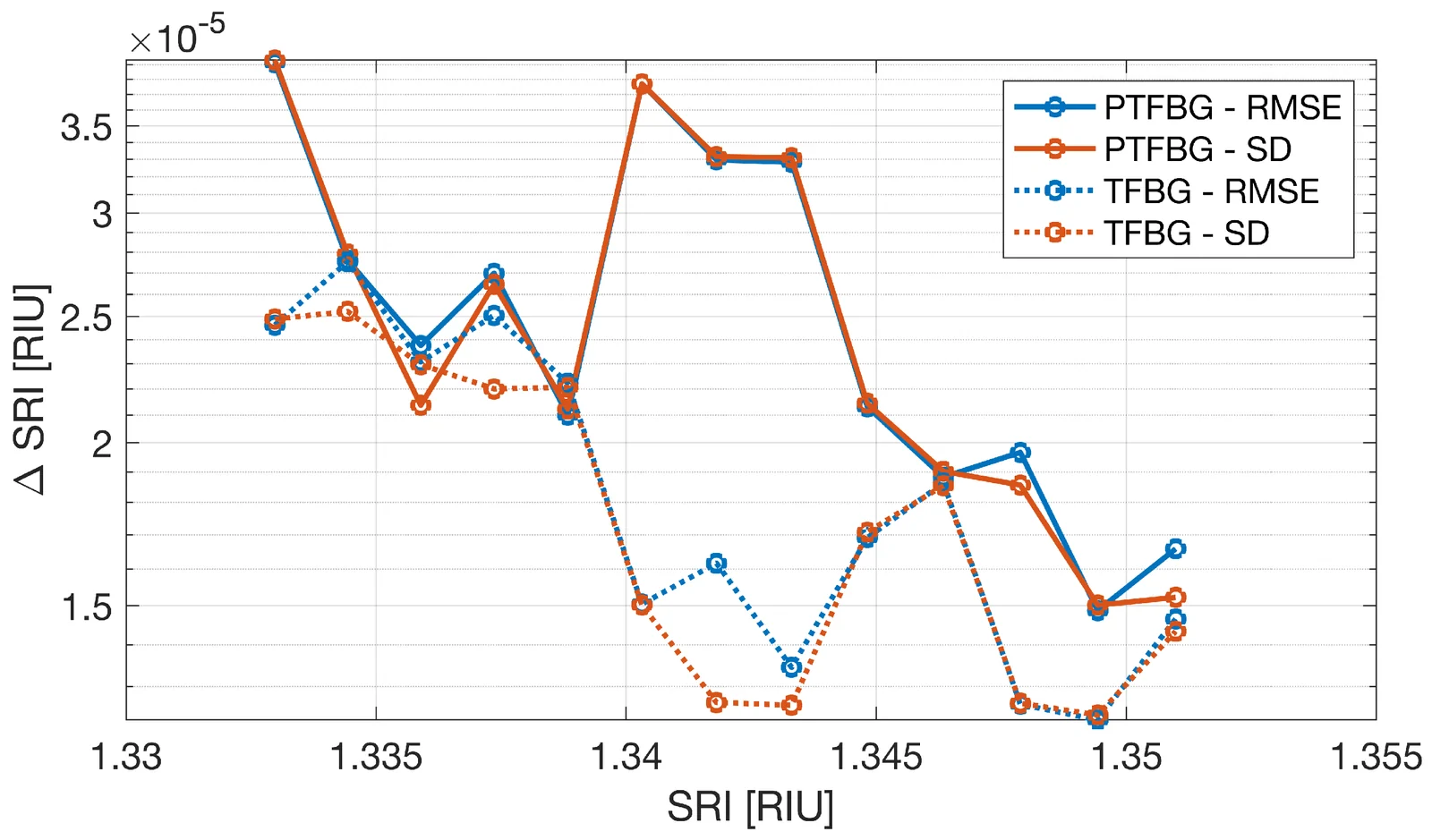
The RMSE and SD values for SRI estimation using a neural network, with random selection of the training, test and validation sets.

SD is the level of dispersion of the results obtained from the NN model:

(2) $$SD=\sqrt{\frac{1}{46}\sum_{i=1}^{46}{\left({SRI}_{i}-\mu \right)}^{2}}$$

where µ is the mean value determined from repeated measurements for all polarizations. The SRI estimation errors for all 598 measured spectra are shown in Figure 13. Since the division of the spectra into training, test and validation sets was random, the training set contains spectra for all 13 SRI values. Consequently, the errors shown in Figure 13 are characterized by high accuracy, and the mean error for each SRI value is close to zero. The data in Figure 14 confirm these observations by showing the SD and RMSE values for the TFBG and PTFBG systems. Furthermore, it should be noted that the RMSE values are lower for both the TFBG and PTFBG systems.

**Figure 15:**
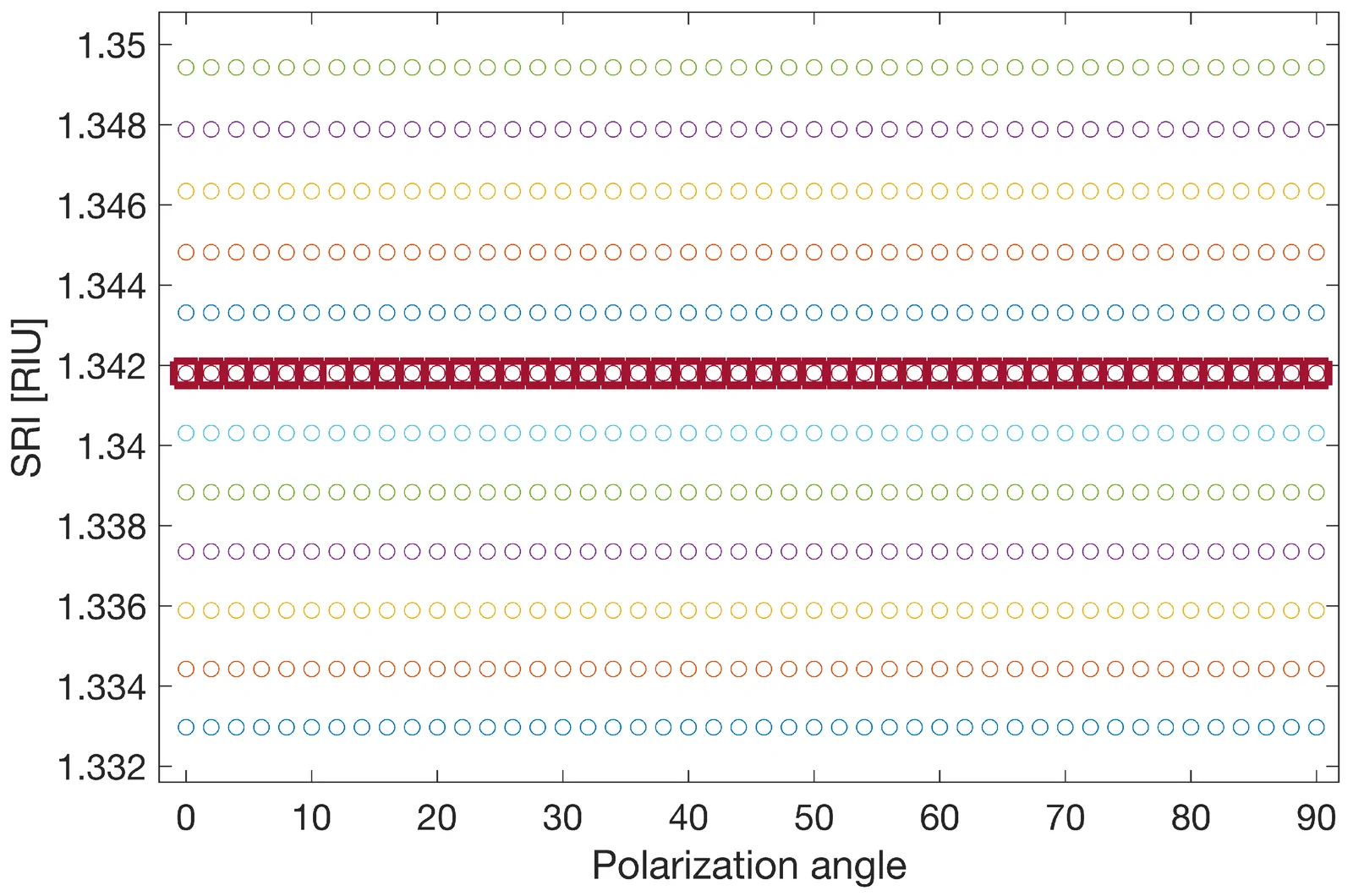
Splitting the dataset for construction and final testing of a single model.

A key consideration in developing and evaluating the model is how the dataset is partitioned. The objective was to estimate SRI from 598 spectra acquired at 13 distinct SRI values, with 46 spectra recorded at different polarization angles for each value. A random split of the entire dataset would allow spectra corresponding to the same SRI value to appear in both the training and test sets. Such a split would not directly assess the model’s ability to estimate SRI values absent from the training data. We therefore adopted a group-based 13-fold cross-validation strategy, with each group containing all spectra corresponding to one SRI value. Because the number of folds equaled the number of groups, this procedure was equivalent to leave-one-group-out cross-validation. In each iteration, 12 SRI groups, comprising 552 spectra, were used for model development, while the remaining group, comprising 46 spectra at different polarization angles, was reserved as the outer test set. The procedure was repeated 13 times, so that each SRI group served as the outer test set once. Within each iteration, the 552 spectra used for model development were randomly divided into training, validation, and internal test sets in proportions of 70%, 15%, and 15%, respectively. These subsets were used for model training, validation, and internal performance assessment. The resulting model was then evaluated on the 46 spectra in the outer test set, which had not been used during model development. This procedure assesses the model’s ability to estimate an SRI value not represented in its development data, across the polarization angles included in the measurements. This assessment is particularly relevant given the limited number of distinct SRI values in the dataset. Figure 15 illustrates the dataset partitioning for a single cross-validation iteration.

**Figure 16:**
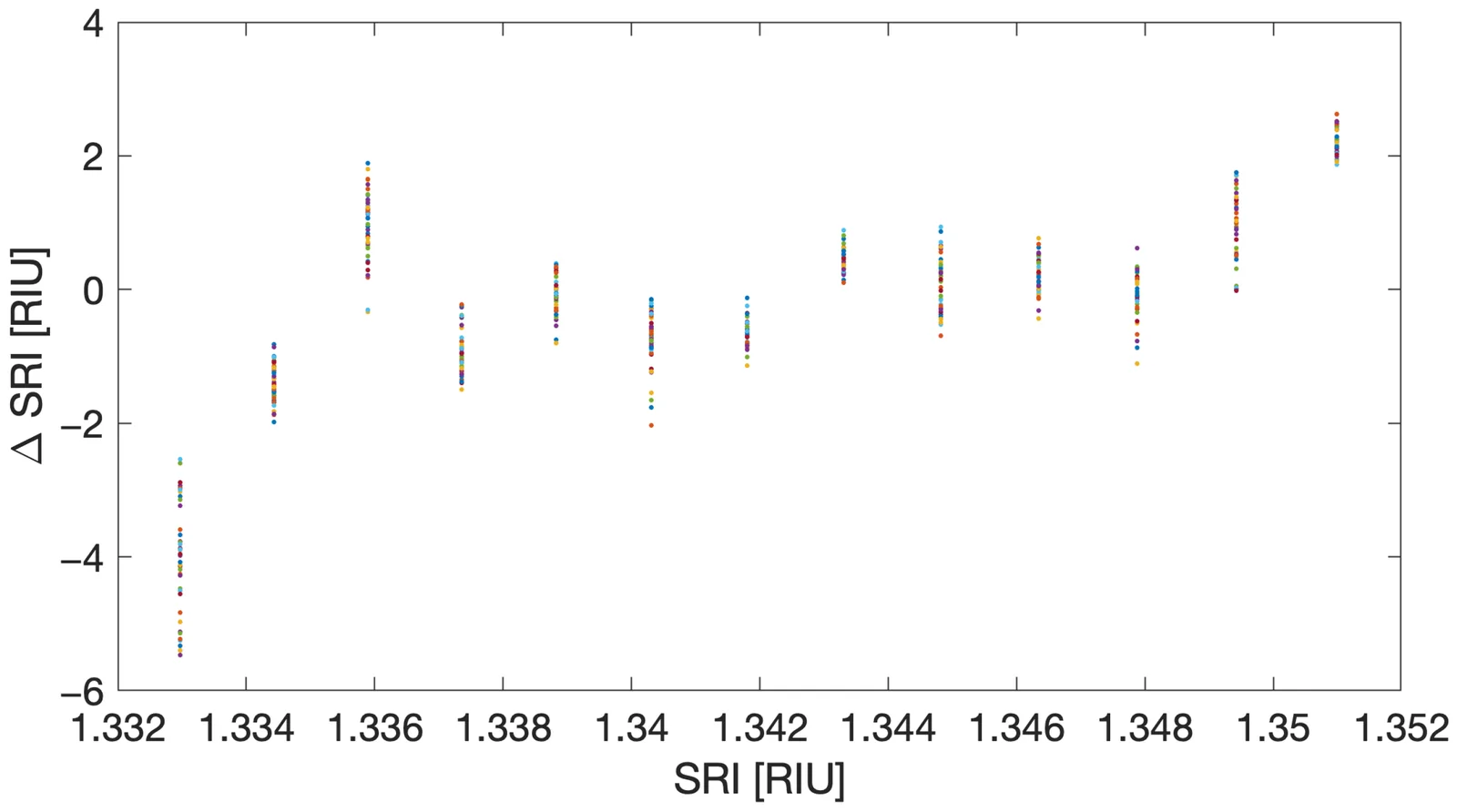
Estimation errors for the SRI values at all measurement points in the case of the TFBG grating and training the network using cross-validation.

Using the correct method to construct and test the network results in a slightly different error distribution than randomly dividing the measurements. As shown in Figure 16, the errors calculated for different polarizations do not oscillate around zero but instead exhibit an additional constant offset. A particularly large offset can be seen at extreme SRI values. This effect occurs because the model lacks information about this region, necessitating extrapolation and consequently increasing the error values.

**Figure 17:**
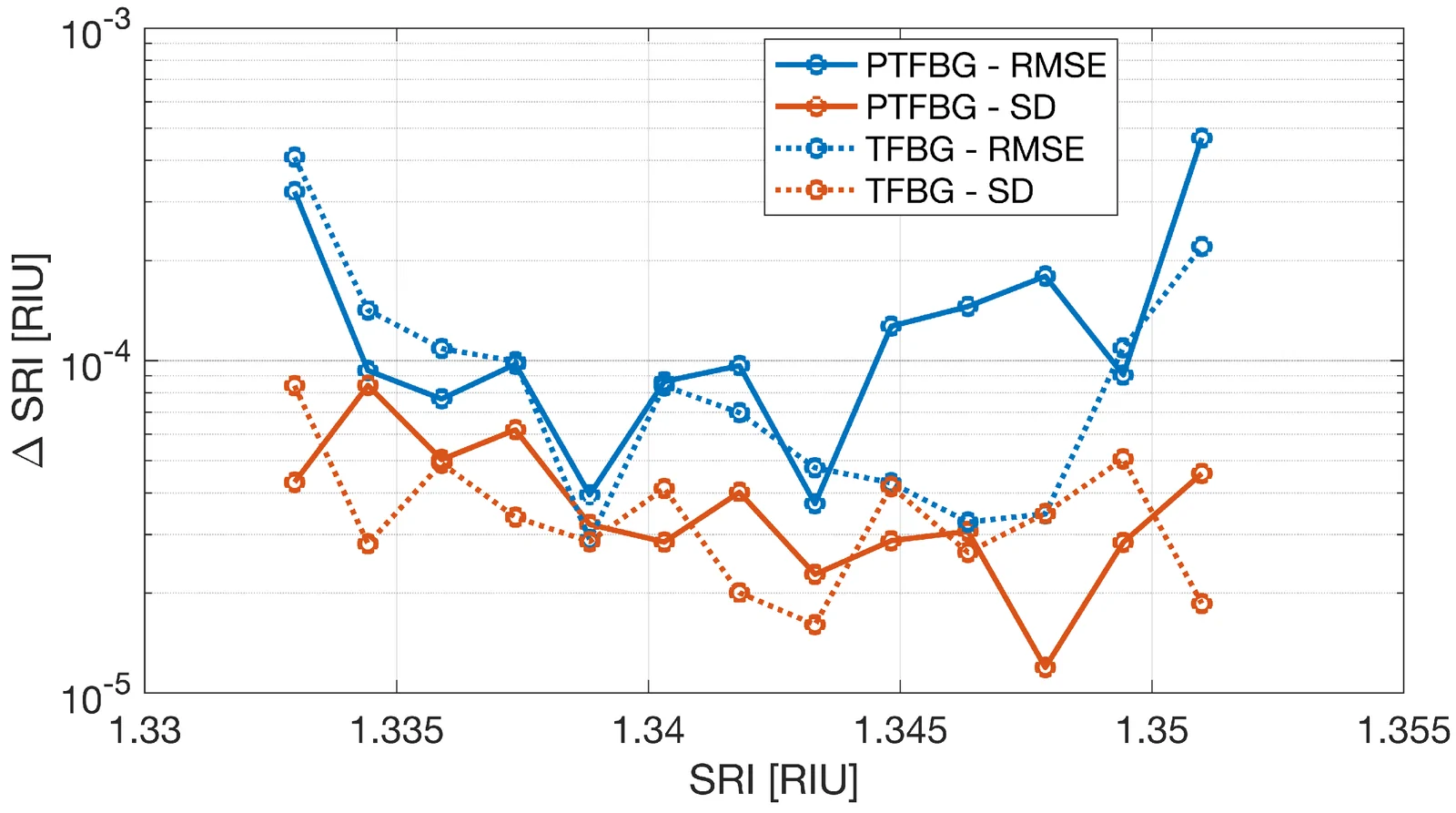
Comparisons of the SD and RMSE metrics for estimating the SRI using a neural network. Input data: Local mode shifts.

As shown in Figure 17, the RMSE values exceed the corresponding SD values. While the SD remains approximately constant across the investigated SRI range, the RMSE is higher near its boundaries. These larger errors may be reduced by including calibration measurements at both ends of the SRI range. Ideally, the calibration range should extend beyond the range anticipated in subsequent measurements. Increasing the number of distinct calibration SRI values near the boundaries may also improve estimation accuracy in these regions.

**Figure 18:**
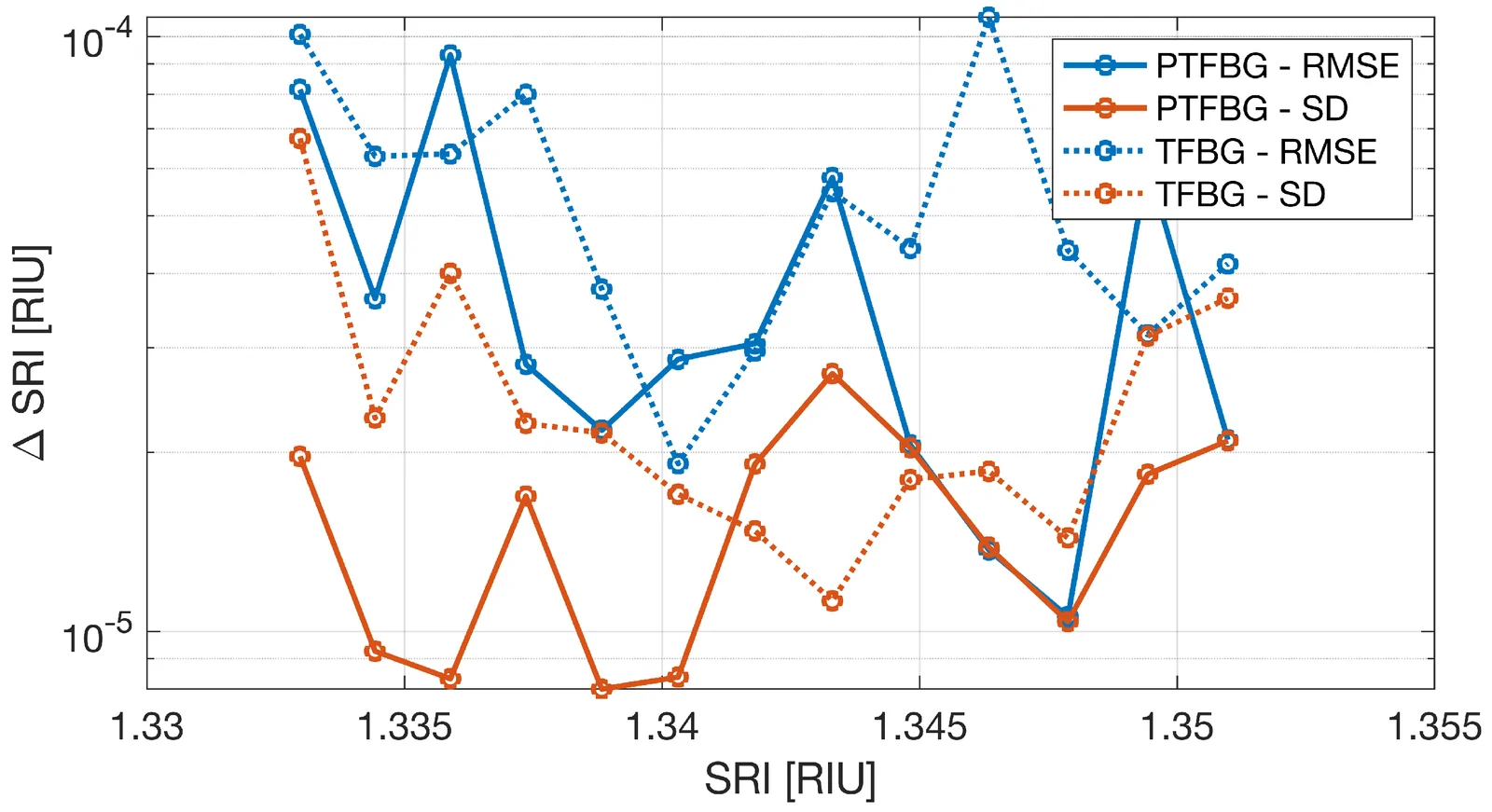
Comparisons of the SD and RMSE parameters for SRI estimation using a neural network. The input data are local mode shifts and cutoff wavelengths.

To make the analyzed demodulation method less sensitive to the polarization of the input light, a cutoff wavelength was added to the feature vector (see Figure 18). This modification resulted in lower RMSE and SD values, whose mean values are presented in Table 1. However, the SD remained uniform across the entire SRI range, whereas the RMSE was greater at the extreme SRI values.

Unfortunately, adding a new feature in the form of the optical spectrum length does not yield any beneficial results. When the first and last SRI values for the TFBG were excluded, the RMSE value decreased from 9.64 × 10^−5^ to 5.50 × 10^−5^ RIU; however, it remained higher than the corresponding value obtained without including the spectral length. The SD value remained at the same level in both cases (TFBG and PTFBG).

## 4. Conclusions

To summarize, we proposed a method for reducing the polarization sensitivity of the TFBG spectrum when determining the refractive index using multimode shifting. Applying the algorithm reduced the RMSE value associated with changes in polarization from 14.00 × 10^−5^ to 5.52 × 10^−5^ RIU for the TFBG grating. Further desensitization was achieved by adding the cutoff wavelength to the input vector. However, including the spectrum length as an input feature provided no benefit; in fact, it worsened the estimation of the SRI. The RMSE and SD values were lower when a PTFBG system was used than when a single TFBG grating was used. Furthermore, we demonstrated that a proper comparison of demodulation methods is possible only through cross-validation. Without an appropriate validation procedure, the model’s performance is overestimated.

## Figures and Tables

**Table 1:** Comparisons of the SD and RMSE values for the different input model parameters for both the TFBG and the PTFBG.

Method	Parameter	TFBG	PTFBG
$\Delta \lambda_{c}$	SD	11.86 × 10^−5^	2.83 × 10^−5^
	RMSE	14.00 × 10^−5^	4.02 × 10^−5^
$NN\left(\Delta \lambda_{m1-15}\right)$	SD	2.58 × 10^−5^	3.91 × 10^−5^
	RMSE	5.52 × 10^−5^	14.30 × 10^−5^
$NN\left(\Delta \lambda_{m1-15},\Delta \lambda_{c}\right)$	SD	2.20 × 10^−5^	1.52 × 10^−5^
	RMSE	7.96 × 10^−5^	3.88 × 10^−5^
$NN\left(\Delta \lambda_{m1-15},\Delta \lambda_{c},CL\right)$	SD	2.25 × 10^−5^	1.94 × 10^−5^
	RMSE	9.64 × 10^−5^	4.35 × 10^−5^

## Data Availability

The data presented in this study are available on request from the corresponding author.

## Abbreviations

The following abbreviations are used in this manuscript:

TFBG	Tilted Fiber Bragg Grating
PTFBG	Perpendicular Tilted Fiber Bragg Grating
RI	Refractive Index
SRI	Surrounding Refractive Index
RIU	Refractive Index Unit
RMSE	Root Mean Square Error
SD	Standard Deviation
NN	Artificial Neural Network

## References

[1] Harasim D. (2022). Polarization-insensitive refractive index measurement using cascaded perpendicular tilted fiber Bragg gratings. Measurement.

[2] Chen X., Xu J., Zhang X., Guo T., Guan B.-O. (2017). Wide range refractive index measurement using a multi-angle tilted fiber Bragg grating. IEEE Photonics Technol. Lett..

[3] Cięszczyk S., Skorupski K., Panas P. (2022). Single- and double-comb tilted Fibre Bragg grating refractive index demodulation methods with Fourier transform pre-processing. Sensors.

[4] Li K., Xiao Y., Liu F., Yang Z., Yan X., Li Z., Guo T., Xiao G.G., Albert J. (2024). Superfine multiresonant fiber grating sensors assisted with silica capillaries. Photonics Res..

[5] Jean-Ruel H., Albert J. (2024). Recent advances and current trends in optical fiber biosensors based on tilted fiber Bragg gratings. TrAC Trends Anal. Chem..

[6] Chan C.-F., Chen C., Jafari A., Laronche A., Thomson D.J., Albert J. (2007). Optical fiber refractometer using narrowband cladding-mode resonance shifts. Appl. Opt..

[7] Chubchev E.D., Dolzhenko E.I., Tomyshev K.A., Nechepurenko I.A., Dorofeenko A.V., Butov O.V. (2025). Analysis of propagation/leakage transition of fiber cladding modes for processing of data from dielectric tilted fiber Bragg grating sensors. Measurement.

[8] Huang Z., Yang N., Xie J., Han X., Yan X., You D., Li K., Vai M.I., Tam K.W., Guo T. (2023). Improving accuracy and sensitivity of a tilted fiber Bragg grating refractometer using cladding mode envelope derivative. J. Light. Technol..

[9] Cięszczyk S., Klimek J., Panas P., Skorupski K., Harasim D. (2026). Determination of SRI values using TFBG and PTFBG based on the wavelength shift of modes, mode groups, and differential methods, considering the influence of polarization. Opt. Express.

[10] Chen S., Yao F., Ren S., Wang G., Huang M. (2022). Cost-effective improvement of the performance of AWG-based FBG wavelength interrogation via a cascaded neural network. Opt. Express.

[11] Wu Y., Xia L., Wu N., Wang Z., Zuo G. (2021). Optimized feedforward neural network for multiplexed extrinsic Fabry–Perot sensors demodulation. J. Light. Technol..

[12] Raju B., Kumar R., Dhanalakshmi S. (2024). Design and implementation of tilted FBG for concurrent temperature and humidity measurement using machine learning. Opt. Fiber Technol..

[13] Leal-Junior A., Avellar L., Frizera A., Caucheteur C., Marques C. (2024). Machine learning approach for automated data analysis in tilted FBGs. Opt. Fiber Technol..

[14] Liu Z., Liu C., Guo T., Li Z., Liu Z. (2025). Deep learning assisted real-time and portable refractometer using a π-phase-shifted tilted fiber Bragg grating sensor. Photonics Res..

[15] Cao Z., Xia T., Zhang S., Zhang S., Mei Y., Liu Z., Guo T., Li Z. (2022). Improved spectral interrogation of tilted fiber Bragg grating refractometer using residual convolutional neural networks. J. Light. Technol..

[16] Imas J.J., Bai X., Zamarreño C.R., Matías I.R., Albert J. (2023). Accurate compensation and prediction of the temperature cross-sensitivity of tilted FBG cladding mode resonances. Appl. Opt..

[17] Gao W., Liu M., Mao J., Wang H., Sun D. (2026). Dual-mode demodulation of fiber-optic strain sensor based on microfiber interferometer cascaded tilted fiber Bragg grating with temperature compensation. Infrared Phys. Technol..

[18] Takeda S.-I., Sato M., Ogasawara T. (2022). Simultaneous measurement of strain and temperature using a tilted fiber Bragg grating. Sens. Actuators A Phys..

[19] Zhong Z., Chen Z., Yan H., Han D. (2026). Temperature sensing based on accumulation of mode shifts in TFBG. IEEE Photonics Technol. Lett..

[20] Kelly-Richard A., Albert J. (2022). Multiresonant analysis improves the limit of detection of tilted fiber Bragg grating refractometers. Opt. Lett..

